# Impacts of COVID-19 Pandemic on the Mental Health of Working Adults in the Czech Republic: Analysis of Self-Report Psychological Data in a One Year Perspective

**DOI:** 10.3390/ejihpe11030079

**Published:** 2021-09-13

**Authors:** Dalibor Kučera, Iva Stuchlíková, Tomáš Mrhálek

**Affiliations:** Faculty of Education, University of South Bohemia, Dukelská 245/9, 370 05 České Budějovice, Czech Republic; stuchl@pf.jcu.cz (I.S.); tmrhalek@pf.jcu.cz (T.M.)

**Keywords:** COVID-19, working adults, mental health, negative indicators, Czech Republic

## Abstract

The article presents research evidence on psychological impacts of the COVID-19 pandemic on the economically active Czech population aged 18–64 (*n* = 1603; 365 men and 1238 women) within a one year perspective. The aim is to describe the differences in the reported mental health indicators (anxiety, depression, and distress) for women and men in the four age groups, two groups with different working statuses (continuation of work/interrupted working status), and between two phases of the epidemic (March to May 2020 and September 2020 to February 2021). The results point to elevated scores of negative mental health indicators (anxiety, depression, and distress) in different subgroups. More negative experiences in a pandemic situation were found, especially in groups of women, people under the age of 35, and among people with interrupted working status. The results also indicate a deterioration of symptoms over time during the epidemic.

## 1. Introduction

The situation associated with the spread of the COVID-19 pandemic has affected the lives of the entire world population. In our study, we focus on the mental health of working adults, that is, the economically active population aged 18 to 64 years, in order to describe the specifics and changes in the context of the COVID-19 pandemic and to interpret these results from an international perspective. The population of working adults deserves attention for many reasons: they are the most represented group in terms of demographic proportions, they often experienced severe economic consequences of pandemic (in loss of income), and they frequently solve additional problems while providing care to other people (taking care of children). In this paper, we describe the mental health of adult women and men from three different perspectives: in relation to their age, their current working status (usual continuation of work/interrupted working status), and the phase of the epidemic in the Czech Republic (within two research time frames).

For the description of mental health, three indicators were chosen: distress, anxiety, and depression. The distress indicator covers the experience of nonspecific psychological distress (van der Westhuizen et al. [1]), and the anxiety and depression indicators describe the severity of a given emotional state (Zigmond and Snaith [2]). These indicators have been used in several similar national and international studies that pointed to risk factors related to mental health in a number of social and population groups and demonstrated noticeable negative effects (cf. meta-analysis of Prati and Mancini [3]; Kunzler et al. [4]). The fear of COVID-19 fear has facilitated the development of psychiatric symptoms such as depression, confusion, stress, and anxiety among people who have never experienced mental illness before [5], and at the same time it has deepened these conditions in the population where these symptoms are already experienced [6,7]. Several studies reported a serious deterioration in the state of mental health throughout the population, especially at elevated levels of distress and anxiety (e.g., Fisher et al. [8]; Al Dhaheri et al. [9]; McCracken [10]; Xiong et al. [11]; Rossi et al. [12]; Talevi et al. [13]; Salari et al. [14]; Gilan et al. [15]; Szabó et al. [16]; Serafini et al. [17]). These findings are also supported in the study of Trnka and Lorencová [18], which points to an intensification of traumatic feelings, fears, and distress in the general Czech population.

In terms of gender, higher rates of negative symptoms have been repeatedly reported in women compared to men. Women show greater severity in symptoms of anxiety, depression, and distress, denoting an increase in the arousal response relative to stress in women, also during COVID-19 [6,8,19,20,21,22,23,24,25,26,27]. For this reason, we work with this variable in our study and perform analyses separately for two groups based on the gender of the participant.

In terms of age, it is often mentioned that although the elderly population is more at risk for COVID-19, studies show a rather low impact on mental health in this population compared to other age groups [28]. In this context, younger adults (18–24 year olds and people under 35 years of 35) are mentioned as the group most affected by mental health problems due to substantial lifestyle changes (restrictions on social life), economic insecurity, or limited experience with crisis situations [8,20,21,22,23,25,29,30].

The situation of working adults is even more complicated in many ways. Negative changes in mental health related to the worsening of economic productivity have been reported [31], as well as increased general psychological distress and anxiety [9,32]. These risks are (again) more pronounced in the female population, as, for example, women often work in positions that are more directly affected by pandemics (see Kniffin et al. [19]).

The long-term impact of the pandemic on mental health has not yet been sufficiently mapped. Some studies report a change in the emotional experience of the population, such as a greater level of anxiety associated with economic worries [33] and generally greater anxiety and distress [32,34]. Further studies demonstrate greater levels of distress, and both depressive symptoms and suicidal thoughts/behaviors increased over time [35].

The situation in the Czech Republic during a pandemic in 2020 to early 2021 was, to some extent, close to the situation in other European countries (e.g., anti-epidemic measures, restrictions, and nonlinearity of the epidemic curve over time). However, there are specifics: In spring 2020 the Czech government took unusually extensive measures and the epidemic significantly slowed; in summer 2020, the measures were relaxed, contributing to the outbreak of the so-called ‘second wave of epidemic’ in autumn 2020. At the beginning of 2021, the Czech Republic showed the highest number of people infected with COVID-19 and the highest mortality (per 100,000 inhabitants) compared to both the EU and other countries around the world [36].

The main goal of the study is the identification of particularly affected groups of the population, which can result in, in addition to other things, better targeting of psychological help and support. The study is based on the hypothesis that these groups, defined by gender, age, and working status, differ from one another in relation to the state of their mental health.

## 2. Materials and Methods

### 2.1. Participants

The sample was obtained by convenience sampling. For both phases, the research was promoted through social networks (Facebook and USB university network) and cooperating institutions (mailing), the research online interface was open to the public, and respondents participated voluntarily without financial reward. The participants (*n* = 1603) provided basic demographic data and completed self-reporting questionnaires measuring their levels of mental distress and anxiety and depression. The sample consists of 365 men and 1238 women (see Table 1) aged 18 to 64 years, and they were economically active at the time of data collection (retirees and students were excluded from the analyses). Due to the data collection method and limitations caused by the epidemic situation [37], the sample was not balanced in terms of age and gender variables, e.g., the sample has a significantly increased proportion of women. The increased participation of women in social science research has been repeatedly documented in a number of studies (Dickinson et al. [38] and McCray et al. [39]). The participants were fully informed about the nature of the research and asked for informed consent before participating. The project was approved by the Ethics Committee of the Faculty of Education of the University of South Bohemia.

### 2.2. Instruments

The Hospital Anxiety and Depression Scale (HADS) questionnaire (Zigmond and Snaith [2]) was used to determine current levels of anxiety and depression. HADS is a 14-item questionnaire developed to identify the presence (possible and probable) of anxiety disorders and depression. It is divided into an Anxiety subscale (HADS-A) and a Depression subscale (HADS-D), both of which contain seven items (ranging from 0 to 21). HADS has been used extensively, and there are several hundreds of studies that use HADS as a research instrument (see Cosco et al. [40]). The Czech version published by Bužgová [41] showed acceptable reliability (Cronbach’s alpha 0.82 and 0.80 for the anxiety and depression scales). In the present study, the Cronbach’s alphas were 0.88 for the anxiety and 0.80 for depression.

The Self-Reporting Questionnaire (SRQ20) was used to measure the current mental distress of the participants. SRQ20 was developed by the World Health Organization (WHO) as a screening tool in primary health care settings [42,43]. The test uses 20 items with dichotomous answers (yes/no) and has been used in many studies focused on quick screening of mental problems in emergency and crisis situations (e.g., Ventevogel et al. [44]; Scholte et al. [45]). In the present study, the test’s Cronbach’s alpha was 0.85.

### 2.3. Procedure

**Figure 1:**
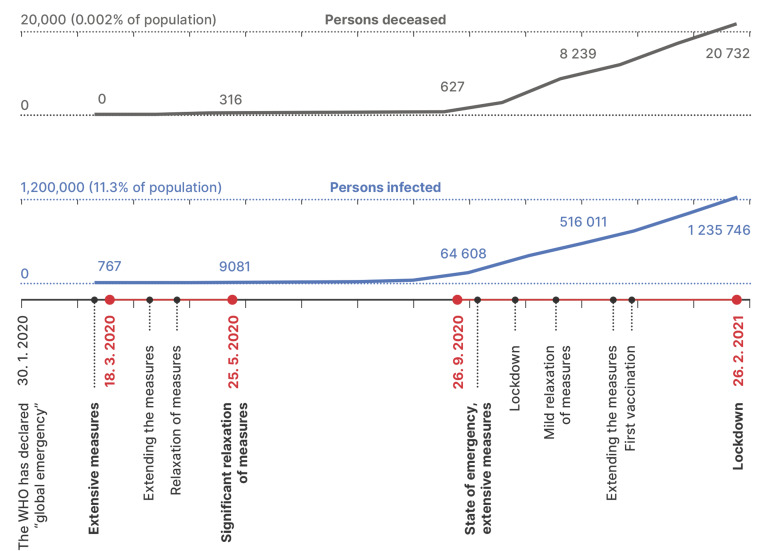
An approximate description of the situation in the Czech Republic during a period of 12 months (two research time frames are highlighted in red; figure created on the basis of official statistics [37]).

Research was carried out as part of the project ‘Research on the psychological effects of the coronavirus epidemic in the Czech Republic’ (JUPSYCOR), which covers two phases of data collection in a one year period, i.e., phases of the epidemic (PE). The first phase (time frame) covered 68 days of the outbreak of the pandemic from 18 March to 25 May 2020 (N1 = 930), and the second phase covered the period of the crisis reescalation from 153 days from 26 September 2020 to 26 February 2021 (N2 = 673). The situational specifics of both time frames, together with the key events that occurred in the Czech Republic, are shown in Figure 1.

### 2.4. Data Analysis

Statistical processing was performed in SPSS 24 for the dependent variables anxiety, depression, and distress in relation to the between-subject factors of gender, current working status (CWS), phase of the epidemic (PE), and age. A high level of correlation was observed between the dependent variables was observed (ρ = 0.72–0.77), which confirms the choice of a multivariate approach. A positively skewed distribution was observed in all dependent variables due to the use of psychiatric instruments among the healthy population; thus, a logarithmic transformation was applied. The assumption of homogeneity of covariance matrices was examined using Box’s test and homogeneity of variance using Levene’s test. Data were analyzed by using general linear models (GLM SPSS), using a factorial design for all independent factors with age as a covariate. Significance levels were established at *p* < 0.05. Post hoc analysis with the Bonferroni multiple comparison test was performed using the estimated marginal means, with age as the covariate evaluated at value 37.84, separately for women and men. In the subsequent analysis that aimed to analyze changes in mental health indicators for subgroups of the population, age was used as a group factor (four age groups were established: 18–25, 26–34, 35–44, and 45–64 years old). The category CWS covered two eventualities: continuation of his/her work or interrupted working status (termination or interruption of a work contract, taking vacation, etc.). The PE category represented two waves of pandemic, i.e., two separate data collections: the first phase is from 18 March to 25 May 2020, and the second phase is from 26 September 2020 to 26 February 2021.

## 3. Results

The sample description and basic descriptives of the anxiety, depression, and distress variables are presented in Table 1.

Multivariate tests using Wilks’ statistic revealed a significant effect on mental health status: Age, Λ = 0.94, F (3, 1952) = 2.56, *p* < 0.01, η = 0.064; Current working status, Λ = 0.99, F(3, 1952) = 6.21, *p* < 0.01, η = 0.012; and Gender, Λ = 0.96, F(3, 1952) = 21, *p* < 0.01, η = 0.038. The phases of the pandemic and the interactions of the factors were not significant with respect to the linear combination of mental health indicators. Tests of between group effects of independent variables on each mental health indicator showed the main effect of gender on all dependent variables: anxiety (F = 51.75, *p* <.01, η = 0.026), depression (F= 14.60, *p* <.01, η = 0.009), and distress (F= 42.64, *p* <.01, η = 0.031). Women scored more negatively on all mental health indicators. Current working status (CWS), i.e., ability or inability to continue working, showed a major effect for anxiety (F = 6.42, *p* < 0.01, η= η = 0.004), depression (F = 18.35, *p* = 0.01, η = 0.011), and distress (F = 7.88, *p* = 0.01, η = 0.005), where the group with interrupted working status reached higher negative symptoms values. Differences according to the phase of the epidemic (PE) were observed in the anxiety level (F = 5.99, *p* = 0.05, η = 0.004), although both distress and depression almost reached the significance level (both *p* = 0.07). The effect of age used as a covariate was significant for distress (F = 64.98, *p* < 0.01, η = 0.039) and for depression (F = 5.84, *p* = 0.02, η = 0.004). The interactions of between subject factors (gender, CWS, and PE) were significant for the ‘Gender*Working status’ interaction in anxiety (F = 3.76, *p* = 0.05, η = 0.002) and depression (F = 3.77, *p* = 0.05, η = 0.002).

A summary of the results, which includes all the main effects tests with the means of the groups and a post hoc comparison of these effects separately for women and men, is provided in Table 2.

Due to differences in mental health indicators between men and women and according to age, post hoc tests were performed separately for both genders with age adjusted as a covariate at value 37.84. Bonferroni’s multiple comparison tests showed a significant worsening in the group of men with interrupted working status (expressed in mean difference, MD) in anxiety (MD = 1.21; CI = 0.33–2.07; *p* = 0.02), depression (MD = 1.24, CI = 0.41–2.05, *p* < 0.01), and distress (MD = 1.03; CI = 0.24–1.83; *p* = 0.02). Women showed deterioration due to interrupted working status only in depression (MD = 0.67; CI = 0.21–1.13; *p* < 0.01). Analysis of the differences between the first and second phases of the pandemic showed a significant deterioration of mental health for women in distress (MD = 0.52; CI = 0.04–0.99; *p* = 0.04) and depression (MD = 0.50; CI = 0.036–0.97; *p* = 0.04).

**Figure 2:**
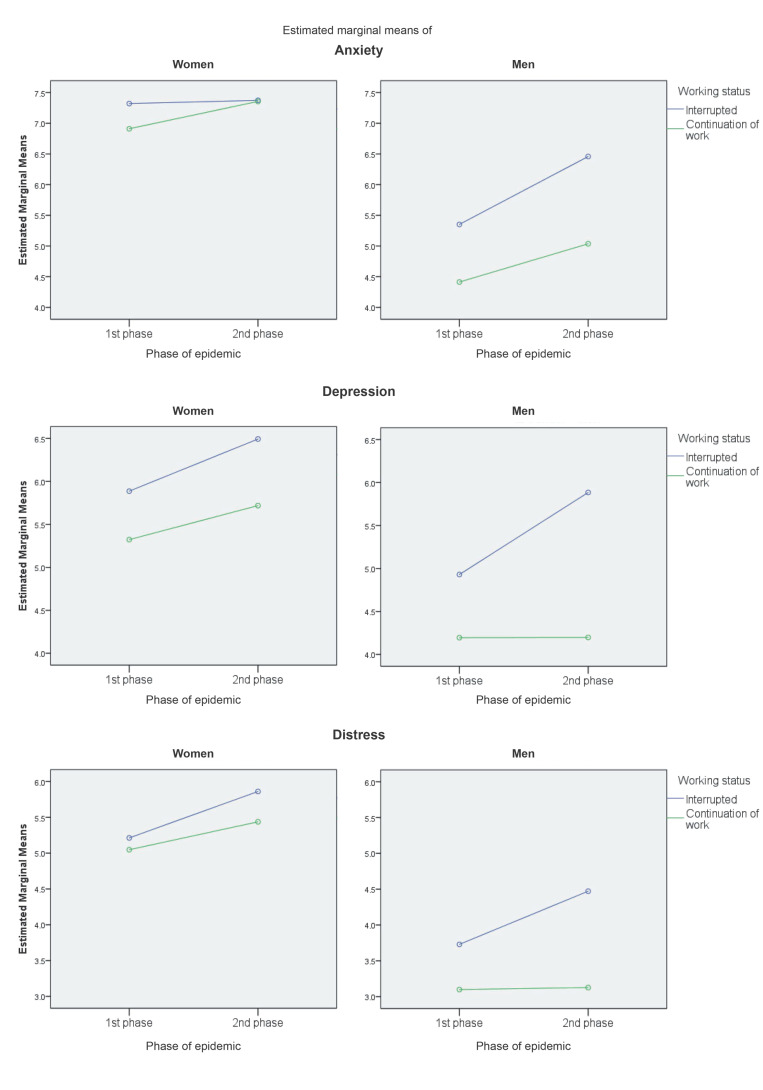
Changes in mental health indicators in women and men between the two phases of epidemic (PE)—differentiated between current working status (CWS) (Note: covariates evaluated at the value Age = 37.84).

**Figure 3:**
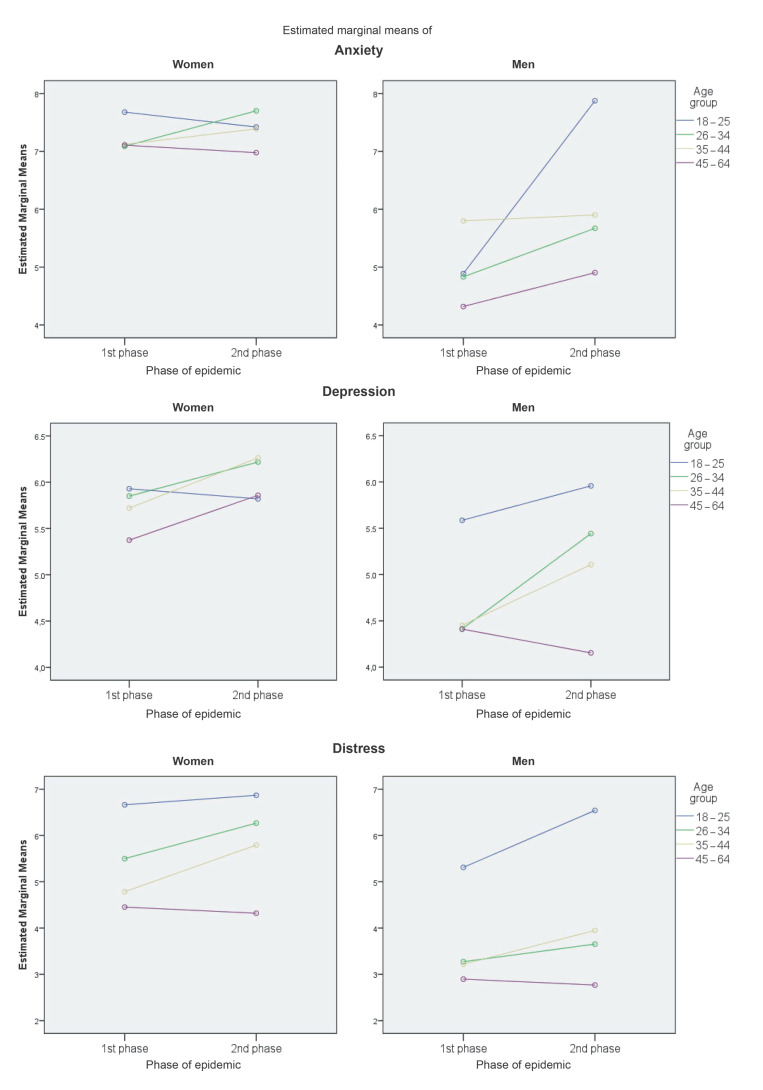
Changes in mental health indicators in women and men between the two phases of the epidemic (PE)—differentiated between four age groups.

In order to explore the effect of age in more detail, the means of mental health indicators for four age groups separately for women and men are presented in Table 2. The pairwise analysis in Figure 2 showed differences between these groups in distress for women (18–25 > ** 35–44, 45–64; 26–34 > ** 45–64) and men (18–25 > ** 26–34, 35–44, 45–64) (* *p* < 0.05; ** *p* < 0.01). Figure 3 illustrates the changes in mental health indicators in two phases of the pandemic according to these four groups. Although the intensity of these changes did reach significance due to multiple comparison corrections only in depression in men aged 18–25 (MD = 2.99, CI = 0.64–5.33. *p* = 0.01), this figure provides a clear visualization of the trends in mental health among age subgroups of the Czech population.

## 4. Discussion

The results showed overall higher scores of negative mental health indicators for the group of women. These findings correspond to current research (e.g., Vindegaard and Benros [6]; Fisher et al. [8]). It is worth mentioning that even outside the pandemic situation, women show higher average scores in negative symptoms than compared to men (e.g., Scholte et al. [45]; Langvik et al. [46]); however, the increase in these scores is particularly pronounced in a pandemic situation (see, e.g., Pieh et al. [20]; Browning et al. [22]; O’Connor et al. [26]). These grounds also justify the importance of carrying out the post hoc analyses separately for a group of women and men.

Current working status (CWS) proved to be an important variable in relation to mental health indicators. Interrupted work status is related to significantly higher levels of anxiety, depression, and distress. The psychological and behavioural consequences associated with working status are also presented in the current study by Yamada et al. [47], and the results are supported by previous research, e.g., Kniffin et al. [19] and Pieh et al. [20].

The age variable was related to stress scores in both women and men, with higher stress levels in the younger population. Respondents younger than 35 years were more depressed, anxious, and stressed. These results are also consistent with previous research, for example, in Australia [8], Saudi Arabia [21], USA [22], Spain [23], and UK [26]. In the group of men, there is also the same direction of significance with respect to the relationship with anxiety, i.e., younger men show higher anxiety scores.

Comparison of mental health indicators between the first phase and the second phase (phases of the epidemic, PE) showed a significant deterioration in mental health, as indicated by score of anxiety. The increase in negative mental health indicators over time is confirmed in previous research (see Savolainen et al. [32]; Veldhuis et al. [35]).

In terms of study limitations, the results could be influenced by local conditions in the Czech Republic (epidemiological, political, or even differences in local seasons and weather). However, given the consensus of our study with the results of many foreign studies, these variables are probably not substantial in terms of the generalizability of the results; similar trends are also evidenced in an international study (48 countries, including the Czech Republic) by Lieberoth et al. [48]. The findings of our study may also be limited by the uneven representation of individual groups, and the smaller proportion of men in the sample also corresponds to a common problem in current research using occasional sampling (underrepresentation of certain population groups). The limitation is also related to the different number of participants within the two phases of data collection (the first phase involved more people) and the different length of the collection time frame (the second phase covered a longer period). Although the first phase of the epidemic was relatively well defined by key public events (beginning with the onset of the epidemic and significant relaxation at the end), the second phase was unfinished in terms of key events at the end of data collection (in February 2021, a new lockdown was ordered by the government).

## 5. Conclusions

Our study points to a significant deterioration in the mental health of specific groups of the working adult population during the COVID-19 epidemic in the Czech Republic. Indicators of anxiety, depression, and distress are scored higher by women and people who cannot currently be regularly present at their workplace (interrupted working status). Throughout the sample, deterioration towards the later stages of the epidemic in anxiety was documented. Given the broad impact of the ongoing pandemic on mental health as presented in the results and the fact that these findings largely correspond to the above-mentioned foreign studies, further research should focus on what factors could facilitate coping with these elevated stress and negative emotion levels within/after the pandemic situation (e.g., social support, flexible responding, strengthening the resilience, and psychological support) and which factors can mitigate the negative impact on vulnerable population groups.

## Figures and Tables

**Table 1:** Sample description, data means (M) and standard deviations (SD) for anxiety and depression (HADS test), and distress (SRQ20 test).

CWS	Age	Phase (PE)		Anxiety				Depression				Distress			
		1st	2nd	PE-1		PE-2		PE-1		PE-2		PE-1		PE-2	
		N	N	M	SD	M	SD	M	SD	M	SD	M	SD	M	SD
**Women**		709	529	7.11	4.45	7.37	4.56	5.59	3.80	5.95	4.14	5.09	4.12	5.59	4.37
CWS-I	18–25	44	22	8.77	4.88	6.91	3.95	6.86	3.22	5.23	4.42	7.55	4.25	6.64	4.76
	26–34	101	48	6.33	4.07	8.04	5.52	5.33	3.19	7.27	4.42	5.16	3.96	6.83	4.60
	35–44	104	45	7.70	4.78	7.24	4.55	6.33	4.28	6.69	4.57	4.94	4.14	5.87	4.63
	45–64	98	45	7.30	4.36	7.04	4.22	5.55	3.85	6.09	4.36	4.45	4.17	4.58	3.89
	Total	347	160	7.32	4.53	7.38	4.68	5.88	3.76	6.49	4.46	5.20	4.20	5.90	4.49
CWS-C	18–25	65	66	6.32	4.47	7.92	4.69	4.89	3.31	6.52	4.07	5.66	4.50	7.20	4.65
	26–34	72	90	7.64	4.66	7.48	4.52	6.13	4.05	5.21	3.67	5.76	4.32	5.90	4.27
	35–44	81	100	6.65	4.32	7.40	4.60	5.22	3.98	5.76	4.49	4.60	3.85	5.49	4.19
	45–64	144	113	6.94	4.21	6.91	4.32	5.14	3.80	5.63	3.66	4.51	3.72	4.06	3.83
	Total	362	369	6.91	4.38	7.36	4.51	5.31	3.82	5.72	3.98	4.99	4.04	5.46	4.31
**Men**		221	144	4.80	3.87	5.47	3.94	4.49	3.64	4.70	3.69	3.40	3.53	3.54	3.67
CWS-I	18–25	10	6	5.20	2.62	9.17	6.65	6.90	3.07	6.00	2.37	6.50	3.10	7.50	5.24
	26–34	31	14	5.26	4.46	6.07	3.83	4.52	3.29	6.64	4.55	3.55	4.06	4.14	2.80
	35–44	21	8	6.14	4.80	6.00	3.66	4.90	3.53	6.25	3.88	3.62	3.93	5.25	3.65
	45–64	22	13	4.95	4.31	6.08	3.50	4.77	3.78	4.92	2.78	3.18	3.91	3.15	3.16
	Total	84	41	5.39	4.29	6.51	4.19	4.96	3.48	5.93	3.59	3.82	3.95	4.54	3.66
CWS-C	18–25	33	12	4.58	3.73	6.58	4.42	4.27	4.15	5.92	4.08	4.12	4.23	5.58	4.85
	26–34	42	37	4.40	3.52	5.27	4.07	4.31	4.09	4.24	3.74	3.00	2.85	3.16	3.46
	35–44	22	28	5.45	3.26	5.36	3.62	4.00	2.83	4.21	3.07	2.82	2.67	2.79	3.01
	45–64	40	26	3.78	3.58	3.73	2.88	4.15	3.48	3.38	3.76	2.65	2.86	2.38	3.52
	Total	137	103	4.43	3.55	5.06	3.77	4.20	3.72	4.21	3.63	3.14	3.23	3.15	3.62

Note: CWS = current working status; I = interrupted working status; C = continuation of work; Phase/PE = phase of the epidemic; N = sample size; M = mean value; SD = standard deviation.

**Table 2:** Summary of results and effects of the main factors: gender, current working status, phase of the epidemic, and age group.

Factor	MHI ^1^	Groups	Est. MM	95% CI		F (df 1)	SMD	Sig.	Gender Effect ^1^
				Lower	Upper				
**Gender** ^2^	Distress **	W	5.40	5.16	5.64	51.75	0.43	0.00	–
		M	3.60	3.15	4.06				
	Anxiety **	W	7.25	6.99	7.51	42.64	0.43	0.00	–
		M	5.33	4.84	5.83				
	Depression **	W	5.86	5.63	6.08	14.60	0.27	0.00	–
		M	4.82	4.38	5.25				
**CWS** ^2^	Distress *	I	4.83	4.41	5.25	7.88	0.16	0.01	* SMD(M) = 0.25
		C	4.17	3.87	4.46				
	Anxiety *	I	6.64	6.19	7.10	6.42	0.16	0.01	** SMD(M) = 0.27
		C	5.94	5.62	6.26				
	Depression **	I	5.81	5.41	6.22	18.35	0.24	0.00	** SMD(W) = 0.17
		C	4.86	4.58	5.14				** SMD(M) = 0.32
**PE** ^2^	Distress	PE-1	4.27	3.96	4.58	3.32	−0.11	0.07	* SMD(W) = 0.12
		PE-2	4.73	4.32	5.14				
	Anxienty *	PE-1	6.01	5.67	6.35	5.99	−0.13	0.02	
		PE-2	6.57	6.13	7.01				
	Depression	PE-1	5.09	4.79	5.39	3.88	−0.13	0.07	** SMD(W) = 0.13
		PE-2	5.58	5.19	5.98				
	**Age Group Factor** ^3^								
**Gender**	**MHI ^1^**	**Age**	**Est. MM**	**95% CI**		**F (df 3)**		**Sig.**	**Pairwise tests ^1^**
				**Lower**	**Upper**				
Women	Distress **	18–25	6.76	6.14	7.38	13.86		0.00	18–25 > 26–34 **, 35–44 **, 45–64 **; 26–35 > 45–64 **
		26–34	5.91	5.45	6.38				
		35–44	5.23	4.77	5.68				
		45–64	4.40	3.97	4.83				
	Anxiety	18–25	7.48	6.81	8.15	0.44		0.73	
		26–34	7.37	6.87	7.88				
		35–44	7.25	6.75	7.75				
		45–64	7.05	6.58	7.52				
	Depression	18–25	5.87	5.28	6.47	0.70		0.55	
		26–34	5.98	5.54	6.43				
		35–44	6.00	5.56	6.44				
		45–64	5.60	5.19	6.02				
Men	Distress **	18–25	5.93	4.71	7.14	7.75		0.00	18–25 > 26–34 **, 35–44 **, 45–64 **
		26–34	3.46	2.69	4.24				
		35–44	3.62	2.63	4.61				
		45–64	2.84	1.99	3.69				
	Anxiety *	18–25	6.38	5.06	7.70	2.22		0.09	
		26–34	5.25	4.41	6.09				
		35–44	5.74	4.66	6.82				
		45–64	4.63	3.71	5.56				
	Depression	18–25	5.77	4.60	6.94	1.55		0.20	
		26–34	4.93	4.18	5.67				
		35–44	4.84	3.89	5.80				
		45–64	4.31	3.49	5.12				

Note: CWS = current working status; I = interrupted working status; C = continuation of work; PE = phase of the epidemic (1st phase/2nd phase); M = men; W = women; MHI = mental health indicators; Est. MM = estimated marginal mean; SMD = standardized mean difference (= mean difference/std. deviation).^1^ Significance * < 0.05, ** < 0.01. ^2^ Covariates are evaluated at the value of Age = 37.84. ^3^ Adjustment for multiple comparisons: Bonferroni FWER.

## Data Availability

Complete research data are freely available on the online repository under the DOI 10.17605/OSF.IO/GQCMD and under the link https://osf.io/gqcmd (accessed on 1 September 2021).
